# Clinical associations of worsening physical function as measured by HAQ-DI scores in systemic sclerosis

**DOI:** 10.1177/23971983251360883

**Published:** 2025-08-19

**Authors:** Jessica L Fairley, Dylan Hansen, Susanna Proudman, Joanne Sahhar, Gene-Siew Ngian, Jennifer Walker, Diane Apostolopoulos, Lauren V Host, Wendy Stevens, Mandana Nikpour, Laura Ross

**Affiliations:** 1 The University of Melbourne, Melbourne, VIC, Australia; 2 St Vincent’s Hospital Melbourne, Melbourne, VIC, Australia; 3 Department of Rheumatology, St Vincent’s Hospital Melbourne, Melbourne, VIC, Australia; 4 The University of Adelaide, Adelaide, SA, Australia; 5 Royal Adelaide Hospital, Adelaide, SA, Australia; 6 Monash Health, Melbourne, VIC, Australia; 7 Monash University, Melbourne, VIC, Australia; 8 Fiona Stanley Hospital, Perth, WA, Australia; 9 The University of Sydney School of Public Health, Sydney, NSW, Australia; 10 SydneyMSK Research Flagship Centre, The University of Sydney, Sydney, NSW, Australia; 11 Royal Prince Alfred Hospital, Sydney, NSW, Australia

**Keywords:** HAQ-DI scores, minimum clinically important difference, physical function, systemic sclerosis

## Abstract

**Background:**

Functional disability is a major concern for individuals with systemic sclerosis (SSc). The Health Assessment Questionnaire–Disability Index (HAQ-DI) measures the ability to perform activities of daily living, with higher scores indicating poorer function.

**Objective:**

To define the frequency and clinical associations of minimum clinically important difference (MCID) change in HAQ-DI scores in SSc.

**Methods:**

Australian Scleroderma Cohort Study participants with two or more HAQ-DI scores ⩽ 2 visits apart were included. Generalised estimating equations were used to model the correlates of the MCID improvement (-0.125 points) and MCID worsening (+0.14 points) of HAQ-DI scores. Subgroup analysis in those with incident (⩽5 years SSc duration at recruitment) and diffuse SSc (dcSSc) were performed.

**Results:**

Of 1117 participants, 712 (64%) recorded worsening of HAQ-DI scores. Of 827 participants with baseline HAQ-DI ⩾ 0.125 units, 585 (71%) had recorded improvement. Across 3229 study visits, older age (odds ratio (OR) 1.1, 95% confidence interval (CI) 1.1–1.1, *p* < 0.01), higher skin score (OR 1.1, 95% CI 1.0–1.2, *p* = 0.01), digital ulcers (OR 1.3, 95% CI 1.0–1.5, *p* = 0.02), raised C-reactive protein (CRP; OR 1.3, 95% CI 1.1–1.6, *p* < 0.01) and patient-reported worsening Raynaud’s phenomenon (RP; OR 1.2, 95% CI 1.0–1.4, *p* = 0.04) and dyspnoea (OR 1.3, 95% CI 1.1–1.6, *p* < 0.01) were associated with worsening of HAQ-DI scores. In those with incident SSc, raised CRP and patient-reported worsening RP and dyspnoea were associated with worsening of HAQ-DI scores. In dcSSc, only raised CRP was associated with worsening HAQ-DI scores, while in lcSSc higher baseline HAQ-DI score, older age, higher skin score, proximal weakness and worsening dyspnoea were associated with worsening scores. Only higher baseline HAQ-DI score was associated with improvement in HAQ-DI scores in the overall cohort (OR 1.3, 95% CI 1.2–1.5, *p* < 0.01).

**Conclusions:**

Two-thirds of a large SSc cohort demonstrated significant change in physical function. Worsening symptom burden and elevated CRP were important determinants of worsening function.

## Graphical abstract


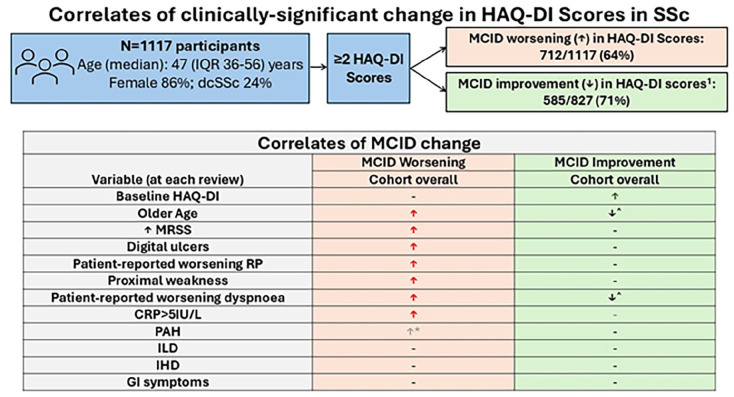


^a^Improvement in HAQ-DI score only able to be assessed in those with a baseline HAQ-DI score ⩾ 0.125 (MCID for improvement).

Abbreviations: ASCS (Australian Scleroderma Cohort Study), ACR/EULAR (American College of Rheumatology/European Alliance of Associations for Rheumatology), HAQ-DI (Health Assessment Questionnaire–Disability Index), MCID (minimum clinically important difference), n (number), SSc (systemic sclerosis).

## Introduction

Functional disability affects more than two-thirds of people living with systemic sclerosis (SSc).^1–3^ Impaired physical function is a major concern for patients and contributes to reduced health-related quality of life.^4,5^ The Health Assessment Questionnaire–Disability Index (HAQ-DI) is a patient-reported measure of physical function, with higher scores indicating poorer functional status.^6^ The HAQ-DI encompasses 8 key domains of daily activities, including dressing, arising, eating, walking, hygiene, reach, grip and activities.^6^ Participants answer 20 questions about their ability to perform these activities of daily living, with each item rated from 0 to 3, with ‘3’ indicating inability to complete the task.^6^ Overall, scores of 0–1 generally represents mild-moderate disability, 1–2 represents moderate to severe disability, and 2–3 represents severe to very severe disability.^7^ The minimum clinically important difference (MCID) in patient-reported outcome measures is defined as the smallest change in score that is meaningful for a patient, and could thus lead the treating clinical to consider a change in treatment.^8,9^

Given the importance of physical function to individuals living with SSc, the HAQ-DI is commonly used as a secondary endpoint in clinical trials, with good retest reliability.^10^ However, modelling suggests HAQ-DI scores tend to be relatively stable over time, raising questions about its widespread utility as an outcome measure in clinical trials.^8,11^ Furthermore, while sensitivity to change has been identified in certain subgroups, for example, diffuse cutaneous SSc (dcSSc),^12^ limited data describe the frequency and correlates of clinically meaningful change in HAQ-DI scores in other SSc subgroups. Accordingly, this study sought to explore the frequency and correlates of clinically meaningful improvement or worsening in HAQ-DI scores over time in SSc.

## Methods

Participants were recruited from the Australian Scleroderma Cohort Study (ASCS), a prospective longitudinal study of SSc. The ASCS has been approved by human research ethics committees at participating sites with St Vincent’s Hospital Melbourne as the coordinating site (HREC-A 020/07). Written informed consent was obtained from all participants. Participants meeting American College of Rheumatology/European Alliance of Associations for Rheumatology (ACR/EULAR) criteria for SSc^13^ recruited between 2007 and June 2024 with a definable disease subclass according to LeRoy criteria (dcSSc or limited (lcSSc))^14^ were included. HAQ-DI scores were recorded annually at study visits. As previously defined in SSc, the MCID used for increasing (worsening) HAQ-DI scores was +0.14 units^15,16^ and MCID for decreasing (improving) HAQ-DI was −0.125.^8^ For inclusion, participants needed to have recorded at least two HAQ-DI Scores during follow-up at a maximum of two visits apart, and a recorded date of SSc onset. Participants were excluded if they had not recorded two HAQ-DI scores during follow-up, or if scores were >2 study visits apart. Incident SSc was defined as ⩽5 years from SSc onset to ASCS recruitment, while prevalent SSc was defined as >5 years from SSc onset to recruitment.^11^

### Clinical data

Demographic and disease data and medication usage were prospectively collected at annual study visits. Disease manifestations were considered present if they were recorded at any time from SSc diagnosis. SSc duration was defined from the time of onset of the first non-Raynaud skin manifestation. Participants were asked at each visit if they had experienced worsening breathlessness or Raynaud’s phenomenon in the month prior to the visit (yes/no). The presence of comorbidities, smoking and medication use were recorded from patient-reported history and medical record review at each study visit. Patient-reported symptoms and clinical examination features were recorded at each visit (including synovitis, tendon friction rubs, and proximal weakness on manual muscle testing (defined as scores <5/5)). Interstitial lung disease (ILD) was diagnosed in the presence of typical radiographic abnormalities on high-resolution computed tomography. Myositis was diagnosed by a positive muscle biopsy. The Medsger Severity Scores were calculated at each visit to assess the overall SSc burden at each study visit.^17^ All participants underwent annual transthoracic echocardiography and pulmonary function testing (including forced vital capacity (FVC) and diffusing capacity for carbon monoxide (DLCO) corrected for haemoglobin) to screen for pulmonary arterial hypertension (PAH) and ILD. Right heart catheterisation or chest high-resolution computed tomography (HRCT) were performed if abnormalities were detected on examination or screening investigations. PAH was defined according to the revised PAH classification criteria^18^ (mean pulmonary artery pressure (mPAP) > 20 millimetres of mercury (mmHg), pulmonary vascular resistance (PVR) > 2 Wood units and a pulmonary arterial wedge pressure (PAWP) ⩽ 15 mmHg), or if PVR unavailable, according to previous classification criteria (mPAP ⩾ 25 mmHg, PAWP < 15 mmHg). Ischaemic heart disease (IHD) was defined as abnormal coronary angiography or patient-reported angina/myocardial infarction. Raised C-reactive protein (CRP) was defined as values > 5 mg/L. Gastrointestinal symptoms were defined as reflux, vomiting, sicca symptoms, diarrhoea/constipation.

### Statistical analysis

Characteristics of study participants are presented as mean (standard deviation (SD)) for normally distributed continuous variables, median (interquartile range (IQR)) for non-normally distributed continuous variables, and as number (percentage) for discrete variables. For pairwise-comparisons of disease features and demographic data, participants were divided into those who recorded MCID worsening of HAQ-DI score at any time, and those who had not. Comparisons between demographics and clinical characteristics between groups were performed using two-sample *t*-test for normally distributed continuous variables, the Wilcoxon rank-sum test for non-normally distributed continuous variables and the chi-square test for discrete variables. Generalised estimating equations (GEE) using an exchangeable correlation structure were used to model longitudinal data to determine the associations of MCID change in HAQ-DI score at each study visit. All eligible MCID measurements (where HAQ-DI scores were ⩽ 2 visits apart) were included. Covariates were chosen for the multivariable analysis if they were either clinically relevant or statistically significant associations of worsening of HAQ-DI scores on univariable analysis (*p* < 0.05) and were not collinear. Collinearity was tested by calculating a Pearson’s correlation coefficient. Variables with a Pearson’s correlation coefficient > 0.30 were considered collinear and the more clinically important variable retained. Participants with missing data were excluded listwise from the model. The same multivariable model was then applied to explore the correlates of clinically significant worsening in HAQ-DI scores in prespecified subgroups (lcSSc vs dcSSc and incident vs prevalent SSc). This model was also applied to identify the correlates of improvement in HAQ-DI scores, after excluding those with baseline HAQ-DI scores <0.125 units who could not record significant improvement in function compared with baseline values. Results are reported as odds ratios (OR) with accompanying 95% confidence intervals (CI). Analysis was performed using STATA 17.0 (Statacorp LP, College Station, TX, USA).

## Results

**Figure 1.**
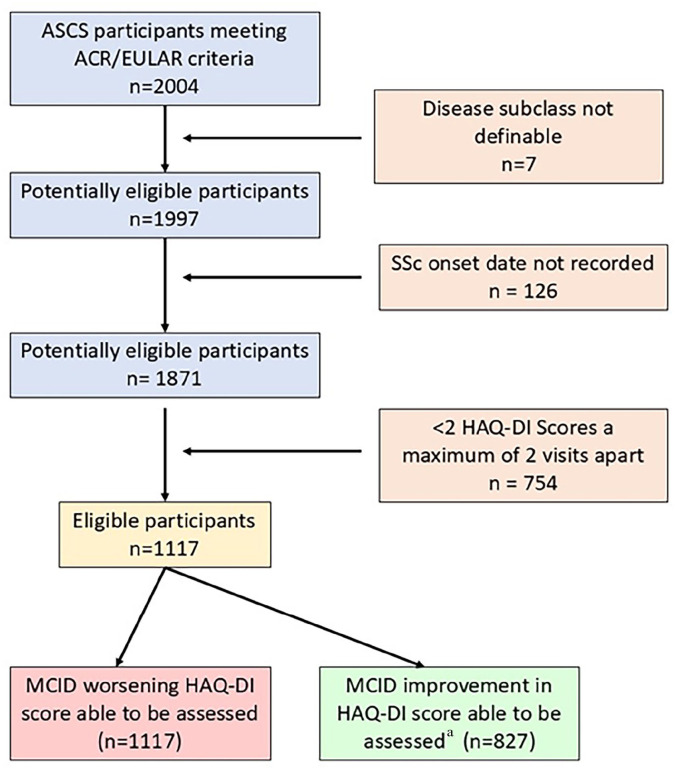
Flowchart of included participants. ^1^MCID for improvement in HAQ-DI score only assessed in those with HAQ-DI scores at baseline⩾ 0.125 units. ^2^Summary of variables in multivariable models involving each subgroup which were associated with a minimum clinically important increase in HAQ-DI Score. ↑Denotes *p*-value of ⩽0.05. ↑* Denotes *p*-value of >0.05 but <0.10. -Denotes *p*-value ⩾ 0.10. ^Denotes variables inversely associated with MCID improvement in HAQ-DI score. Abbreviations: CRP (C-reactive protein), dcSSc (diffuse cutaneous SSc), GI (gastrointestinal), HAQ-DI (Health Assessment Questionnaire–Disability Index), IHD (ischaemic heart disease), ILD (interstitial lung disease), IQR (interquartile range), lcSSc (limited cutaneous SSc), MCID (minimum clinically important difference), MRSS (modified Rodnan Skin Score), N (number), PAH (pulmonary arterial hypertension), SSc (systemic sclerosis).

One-thousand one-hundred and seventeen ASCS participants recorded at least two HAQ-DI scores a maximum of two annual study visits apart and were included (Figure 1; Supplementary Figure S1). Participants recorded a median number of 4 HAQ-DI scores (IQR 3–7 scores). Among included participants, 712 (64%) recorded a MCID worsening in HAQ-DI score (Table 1). Of 827 participants with a baseline HAQ-DI score ⩾ 0.125 units where improvement could be examined, 712/827 (71%) recorded a MCID reduction (improvement) in HAQ-DI score (Figure 1). Overall, 423 participants (51%) had recorded both a significant improvement and worsening, 325 (39%) had recorded either improvement or worsening but not both, and 79 (10%) had recorded neither improvement nor worsening.

**Table 1.** Demographic characteristics of the included cohort.

Variable	Whole cohort (n = 1117)	MCID worsening of HAQ-DI score* (n = 712, 64%)	Stable HAQ-DI score* (n = 405, 36%)	*p*-value
Age at SSc onset (n = 1116)	47.2 (36.0–56.4)	47.6 (37.0–56.8)	45.7 (34.5–55.7)	0.12
Female sex (n = 1117)	961 (86.0%)	622 (87.4%)	339 (83.7%)	0.09
dcSSc (n = 1117)	273 (24.4%)	182 (25.6%)	91 (22.5%)	0.25
MRSS (highest recorded) (n = 1115)	8 (5–15)	9 (5–17)	7 (4–13)	<0.01
Follow up (years) (n = 1117)	6.9 (4.0–10.4)	7.6 (4.6–11.2)	5.4 (3.0–9.0)	<0.01
HAQ-DI score (baseline) (n = 1117)	0.5 (0–1.1)	0.6 (0.3–1.3)	0.25 (0–0.9)	<0.01
Number of HAQ-DI scores recorded (n = 1117)	4 (3–7)	5 (3–8)	3 (2–4)	<0.01
Medsger Severity Score (Baseline) (n = 1117)	5 (3–7)	6 (4–8)	4 (3–6)	<0.01
Smoked* (n = 1117)	582 (52.1%)	375 (52.7%)	207 (51.1%)	0.62
ANA Centromere (n = 1140)	508 (45.6%)	334 (47.0%)	174 (43.2%)	0.22
ENA Scl-70 (n = 1107)	166 (15.0%)	113 (16.0%)	53 (13.3%)	0.22
RNA Polymerase-3 (n = 861)	111 (12.9%)	83 (14.8%)	28 (9.4%)	0.02
PAH* (n = 1117)	136 (12.2%)	105 (14.7%)	31 (7.7%)	<0.01
IHD*^1^ (n = 1117)	179 (16.0%)	133 (18.7%)	46 (11.4%)	<0.01
ILD*^2^ (n = 1117)	344 (30.8%)	241 (33.8%)	103 (25.4%)	<0.01
FVC < 80%* (n = 1104)	398 (36.1%)	289 (41.1%)	109 (27.2%)	<0.01
DLCO < 80%* (n = 1072)	709 (66.1%)	510 (74.2%)	199 (51.7%)	<0.01
Gastrointestinal involvement*^3^ (n = 1117)	1,088 (97.4%)	705 (99.0%)	383 (94.6%)	<0.01
Digital Ulcers* (n = 1117)	638 (57.1%)	454 (63.8%)	184 (45%)	<0.01
Myositis (biopsy-proven)* (n = 1117)	35 (3.1%)	21 (2.9%)	14 (3.5%)	0.64
Proximal weakness*^4^ (n = 1114)	303 (27.2%)	240 (33.8%)	63 (15.6%)	<0.01
Muscle atrophy* (n = 1113)	280 (25.1%)	216 (30.3%)	64 (15.8%)	<0.01
Synovitis* (n = 1117)	545 (48.8%)	370 (52.0%)	175 (43.2%)	<0.01
Tendon friction rub* (n = 1113)	128 (11.5%)	94 (13.2%)	34 (8.4%)	<0.01
Prednisolone* (n = 1117)	574 (51.4%)	398 (55.9%)	176 (43.5%)	<0.01
Non-corticosteroid Immunosuppression*^5^ (n = 1117)	571 (51.1%)	384 (53.9%)	187 (46.2%)	0.01

Abbreviations: CI (confidence interval), dcSSc (diffuse cutaneous systemic sclerosis), GI (gastrointestinal), IHD (ischaemic heart disease), ILD (interstitial lung disease), MRSS (Modified Rodnan Skin Score), PAH (pulmonary arterial hypertension), SSc (systemic sclerosis).

1 IHD defined by composite endpoint of patient-reported angina or acute myocardial infarction, or abnormal coronary angiogram. ^2^ILD diagnosed on lung high-resolution computed tomography. ^3^Gastrointestinal symptoms include history of Barret’s oesophagus, GAVE, oesophageal dysmotility, oesophageal strictures, dysphagia, reflux, vomiting, bowel dysmotility, pseudo-obstruction, constipation, faecal incontinence, diarrhoea or bloating. ^4^Proximal weakness defined as power on manual muscle testing <5/5. ^5^Non-corticosteroid immunosuppressive treatment defined as ever receiving synthetic or biologic disease-modifying antirheumatic drugs. *Denotes ever from SSc onset.

### Associations of MCID worsening of HAQ-DI scores in the overall cohort

There was no difference in age, ethnicity or SSc subclass between those who had recorded worsening HAQ-DI scores and those who had not (Table 1). Those with worsening scores had a longer duration of follow-up (*p* < 0.01) and had recorded more HAQ-DI scores (5 (3–8) vs 3 (2–4) scores, *p* < 0.01). Higher peak modified Rodnan Skin Scores (MRSS) and baseline Medsger severity scores were recorded in those with a significant increase in HAQ-DI score (both *p* < 0.01). Cardiopulmonary disease including PAH (15% vs 8%, *p* < 0.01), IHD (19% vs 11%, *p* < 0.01), ILD (34% vs 25%, *p* < 0.01), FVC < 80% (41% vs 27%, *p* < 0.01) and DLCO < 80% (74% vs 52%, *p* < 0.01) was more common in those recording worsening in HAQ-DI scores. Gastrointestinal involvement (99% vs 95%, *p* < 0.01), digital ulcers (64% vs 45%, *p* < 0.01), synovitis (52% vs 43%, *p* < 0.01) and tendon friction rubs (13% vs 8%, *p* < 0.01) were more common in this group, as was proximal muscle weakness (34% vs 16%, *p* < 0.01) and muscle atrophy (30% vs 16%, *p* < 0.01) although not myositis (*p* = 0.64). Exposure to prednisolone (56% vs 44%, *p* < 0.01) and non-corticosteroid immunosuppression (54% vs 46%, *p* = 0.01) were also more common in those with a MCID worsening in HAQ-DI score.

In longitudinal multivariable modelling, older age at each review (OR 1.1, 95% CI 1.1–1.1, *p* < 0.01) and higher MRSS (OR 1.1, 95% CI 1.0–1.2, *p* = 0.01) were associated with worsening in HAQ-DI scores between visits (Table 2; univariable analyses in Supplementary Table S1), as were digital ulcers (OR 1.3, 95% CI 1.0–1.5, *p* = 0.02) and patient-reported worsening Raynaud’s phenomenon (OR 1.2, 95% CI 1.0–1.4, *p* = 0.04). Raised CRP (OR 1.3, 95% CI 1.1–1.6, *p* < 0.01) and worsening dyspnoea (OR 1.3, 95% CI 1.1–1.6, *p* < 0.01) were associated with worsening in HAQ-DI scores, as was PAH (OR 1.3, 95% CI 1.0–1.6, *p* = 0.08) although not meeting statistical significance. Baseline HAQ-DI score, ILD, IHD and gastrointestinal symptoms were not associated with worsening in HAQ-DI scores (*p* > 0.05).

**Table 2.** Multivariable GEE model for associations of clinically significant worsening of HAQ-DI scores (N = 3229 visits).

Variable (at each study visit)	Odds ratio	95% confidence interval	*p*-value
Baseline HAQ-DI Score	1.1	1.0–1.2	0.22
Age at each review (5-year increments)	1.1	1.1–1.1	<0.01
MRSS (5-point increments)	1.1	1.0–1.2	0.01
Digital ulcers at each visit	1.3	1.0–1.5	0.02
Worsening Raynaud’s Phenomenon^1^	1.2	1.0–1.4	0.04
Proximal weakness^2^	1.4	1.1–1.9	0.02
PAH	1.3	1.0–1.6	0.08
ILD^3^	1.0	0.8–1.4	0.96
IHD^4^	1.2	0.8–1.6	0.39
Worsening dyspnoea^4^	1.3	1.1–1.6	<0.01
CRP > 5 mg/L	1.3	1.1–1.6	<0.01
Gastrointestinal symptoms^5^	1.2	0.9–1.5	0.27

Abbreviations: CRP (C-reactive protein), mg/L (milligram/litre), MRSS (modified Rodnan skin score), N (number), PAH (pulmonary arterial hypertension), SSc (systemic sclerosis). ^1^Worsening Raynaud’s phenomenon defined as increasing symptoms in the month prior to review. ^2^Proximal weakness defined as proximal muscle power of <5/5 on manual muscle testing at each study visit. ^3^ILD defined on high-resolution computed tomography of the chest. ^4^IHD defined as patient-reported angina, myocardial infarction or abnormal coronary angiogram. ^5^Worsening dyspnoea at each study visit defined as patient-reported worsening of breathlessness (yes/no) in month prior to study visit. ^6^Gastrointestinal symptoms include dysphagia, reflux, vomiting, gastric antral vascular ectasia, bowel dysmotility, pseudo-obstruction, constipation, faecal incontinence, diarrhoea or bloating.

### Factors associated with a MCID worsening of HAQ-DI scores in incident vs prevalent SSc

In those with incident SSc, 263/437 (60%) had recorded worsening in HAQ-DI score, compared with 449/680 (66%) of those with prevalent SSc (*p* = 0.05). In both incident and prevalent SSc (Table 3), older age (incident OR 1.1, 95% 1.1–1.2, *p* < 0.01; prevalent OR 1.1, 95% CI 1.0–1.1, *p* < 0.01) was associated with worsening of HAQ-DI scores, as was higher MRSS although not meeting statistical significance in incident SSc (incident OR 1.1, 95% CI 1.0–1.2, *p* = 0.08; prevalent OR 1.1, 95% CI 1.0–1.2, *p* = 0.05). In incident SSc only, patient-reported worsening Raynaud’s phenomenon (OR 1.3, 95% CI 1.0–1.8, *p* = 0.05), raised CRP (OR 1.7, 95% CI 1.2–2.2, *p* < 0.01) and PAH (OR 1.8, 95% CI 1.2–2.9, *p* = 0.01) were associated with worsening of HAQ-DI scores, as were digital ulcers although not meeting statistical significance (OR 1.3, 95% CI 1.0–1.8, *p* = 0.08). In prevalent SSc, proximal weakness (OR 1.6, 95% CI 1.1–2.3, *p* = 0.01) and patient-reported worsening dyspnoea (OR 1.5, 95% CI 1.2–1.9, *p* < 0.01) were associated with worsening of HAQ-DI scores. Baseline HAQ-DI score, ILD, IHD and gastrointestinal symptoms were not associated with worsening of HAQ-DI scores in either cohort (*p* > 0.05).

**Table 3.** Subgroup analysis: comparison of disease features associated with worsening HAQ-DI scores at each visit in incident and prevalent SSc (Model A) and limited vs diffuse cutaneous SSc (Model B).

Model A: associations of MCID worsening HAQ-DI scores in incident vs. prevalent SSc						
Variable (at each study visit)	Incident SSc only (N = 1273 observations)			Prevalent SSc only (N = 1956 observations)		
	Odds ratio	95% CI	*p*-value	Odds ratio	95% CI	*p*-value
Baseline HAQ-DI score	1.0	0.8–1.2	0.83	1.2	1.0–1.4	0.11
Age at each review (5-year increments)	1.1	1.1–1.2	<0.01	1.1	1.0–1.1	0.01
MRSS (5-point increments)	1.1	1.0–1.2	0.08	1.1	1.0–1.2	0.05
Digital ulcers at each visit	1.3	1.0–1.8	0.08	1.2	1.0–1.6	0.10
Worsening Raynaud’s Phenomenon^1^	1.3	1.0–1.8	0.05	1.1	0.9–1.4	0.29
Proximal weakness^2^	1.3	0.8–2.1	0.30	1.6	1.1–2.3	0.01
PAH	1.8	1.2–2.9	<0.01	1.1	0.8–1.5	0.58
ILD^3^	0.9	0.5–1.4	0.56	1.1	0.7–1.6	0.76
IHD^4^	1.1	0.6–2.1	0.80	1.2	0.8–1.8	0.42
Worsening dyspnoea^5^	1.0	0.7–1.5	0.93	1.5	1.2–1.9	<0.01
CRP > 5 mg/L	1.7	1.2–2.2	<0.01	1.2	0.9–1.5	0.18
Gastrointestinal symptoms^6^	1.2	0.8–1.7	0.37	1.2	0.8–1.7	0.40
Model B: associations of MCID worsening HAQ-DI scores in dcSSc vs. lcSSc.						
Variable (at each study visit)	dcSSc only (N = 728 observations)			lcSSc only (N = 2501 observations)		
	Odds ratio	95% CI	*p*-value	Odds ratio	95% CI	*p*-value
Baseline HAQ-DI Score	0.9	0.7–1.1	0.34	1.2	1.1–1.5	0.01
Age at each review (5-year increments)	1.0	0.9–1.1	0.70	1.1	1.1–1.2	<0.01
MRSS (5-point increments)	1.1	1.0–1.2	0.07	1.2	1.0–1.3	0.02
Digital ulcers at each visit	1.4	1.0–1.9	0.06	1.2	1.0–1.6	0.11
Worsening Raynaud’s Phenomenon^1^	1.4	1.0–2.0	0.07	1.2	0.9–1.4	0.16
Proximal weakness^2^	1.1	0.7–1.9	0.63	1.7	1.2–2.4	<0.01
PAH	1.4	0.8–2.2	0.21	1.2	0.9–1.7	0.20
ILD^3^	0.9	0.5–1.4	0.53	1.2	0.8–1.8	0.30
IHD^4^	0.9	0.4–2.2	0.86	1.2	0.8–1.7	0.37
Worsening dyspnoea^5^	1.1	0.8–1.7	0.54	1.4	1.1–1.7	<0.01
CRP > 5 mg/L	1.7	1.2–2.4	<0.01	1.2	1.0–1.5	0.07
Gastrointestinal symptoms^6^	0.9	0.5–1.4	0.55	1.2	0.9–1.6	0.17

Abbreviations: CI (confidence interval). CRP (C-reactive protein), mg/L (milligram/litre), MRSS (modified Rodnan skin score), N (number), PAH (pulmonary arterial hypertension), SSc (systemic sclerosis). ^1^Worsening Raynaud’s phenomenon defined as increasing symptoms in the month prior to review. ^2^Proximal weakness defined as proximal muscle power of <5/5 on manual muscle testing at each study visit. ^3^ILD defined on high-resolution computed tomography of the chest. ^4^IHD defined as patient-reported angina, myocardial infarction or abnormal coronary angiogram. ^5^Worsening dyspnoea at each study visit defined as patient-reported worsening of breathlessness (yes/no) in month prior to study visit. ^6^Gastrointestinal symptoms include dysphagia, reflux, vomiting, gastric antral vascular ectasia, bowel dysmotility, pseudo-obstruction, constipation, faecal incontinence, diarrhoea, or bloating.

### Factors associated with MCID worsening HAQ-DI scores in dcSSc vs lcSSc

In those with dcSSc, 182/273 (67%) had recorded worsening of HAQ-DI scores, compared with 530/844 (63%) of those with lcSSc (*p* = 0.25). Raised CRP was associated with worsening of HAQ-DI scores in dcSSc (OR 1.7, 95% CI 1.2–2.4, *p* < 0.01) as well as in lcSSc although not meeting statistical significance (OR 1.2, 95% CI 1.0–1.5, *p* = 0.07) (Table 3). Higher MRSS scores were associated with worsening in HAQ-DI scores in lcSSc (OR 1.2, 95% CI 1.0–1.3, *p* = 0.02), and in dcSSc (OR 1.1, 95% CI 1.0–1.2, *p* = 0.07) although not meeting statistical significance. In lcSSc, baseline HAQ-DI score (OR 1.2, 95% CI 1.1–1.5, *p* = 0.01), older age (OR 1.1, 95% CI 1.1–1.2, *p* < 0.01), proximal weakness (OR 1.7, 95% CI 1.2–2.4, *p* < 0.01) and patient-reported worsening dyspnoea (OR 1.4, 95% CI 1.1–1.7, *p* < 0.01) were associated with worsening of HAQ-DI scores. PAH, ILD, IHD and gastrointestinal symptoms were not associated with worsening of HAQ-DI scores.

### MCID improvement in HAQ-DI scores

Correlates of MCID improvement in HAQ-DI scores were explored after excluding participants with a baseline HAQ-DI < 0.125 units (n = 290, 26%) (Figure 1 and Table 4). Among 827 participants, 585/827 (71%) recorded improvement in HAQ-DI score. In multivariable modelling, only higher baseline HAQ-DI score (OR 1.3, 95% CI 1.2–1.5, *p* < 0.01) was positively associated with improvement in HAQ-DI scores (Table 4). Older age (OR 1.0, 95% CI 0.9–1.0, *p* = 0.02) and patient-reported worsening dyspnoea (OR 0.7, 95% CI 0.6–0.9, *p* < 0.01) were inversely associated with improvement in HAQ-DI scores, indicating that lower likelihood of recording improvement in HAQ-DI scores.

**Table 4.** Multivariable GEE model for associations of MCID improvement in HAQ-DI scores, after excluding those with a baseline HAQ-DI scores < 0.125 units who could not record improvement^1^ (n = 2330 observations).

Variable (at each study visit)	Odds ratio	95% confidence interval	*p*-value
Baseline HAQ-DI score	1.3	1.2–1.5	< 0.01
Age at each review (5-year increments)	1.0	0.9–1.0	0.02
MRSS (5-point increments)	1.0	0.9–1.0	0.13
Digital ulcers at each visit	0.9	0.7–1.1	0.18
Worsening Raynaud’s Phenomenon^1^	1.0	0.9–1.2	0.72
Proximal weakness^2^	0.8	0.6–1.1	0.22
PAH	1.0	0.8–1.3	0.87
ILD^3^	1.0	0.7–1.4	0.99
IHD^4^	1.2	0.8–1.7	0.39
Worsening dyspnoea^4^	0.7	0.6–0.9	< 0.01
CRP > 5 mg/L	0.9	0.8–1.1	0.46
Gastrointestinal symptoms^5^	1.1	0.8–1.5	0.45

1 Of 1117, 290 participants (26%) excluded due to baseline HAQ-DI scores less than 0.125. Abbreviations: CRP (C-reactive protein), mg/L (mg/litre), MRSS (modified Rodnan skin score), N (number), PAH (pulmonary arterial hypertension), SSc (systemic sclerosis). ^1^Worsening Raynaud’s phenomenon defined as increasing symptoms in the month prior to review. ^2^Proximal weakness defined as proximal muscle power of < 5/5 on manual muscle testing at each study visit. ^3^ILD defined on high-resolution computed tomography of the chest. ^4^IHD defined as patient-reported angina, myocardial infarction or abnormal coronary angiogram. ^5^Worsening dyspnoea at each study visit defined as patient-reported worsening of breathlessness (yes/no) in month prior to study visit. ^6^Gastrointestinal symptoms include dysphagia, reflux, vomiting, gastric antral vascular ectasia, bowel dysmotility, pseudo-obstruction, constipation, faecal incontinence, diarrhoea or bloating.

## Discussion

In a large SSc cohort with serial HAQ-DI score measurements and almost 7 years of follow-up, 64% of patients recorded a clinically significant worsening in physical function between study visits. Furthermore, over 70% of participants with a baseline HAQ-DI ⩾ 0.125 units had improvement in their HAQ-DI scores during follow-up, with only 10% of participants recording no clinically meaningful change in HAQ-DI score during follow-up. Together, these data suggest that despite modelling that HAQ-DI trajectories tend to be stable over time in SSc across groups of patients, clinically meaningful change does occur at an individual patient level and there are important clinical correlates of decline in patient function.^11,19^

This study identified important similarities and differences in the clinical associations with clinically meaningful change in physical function across different SSc subgroups. Older age was an important contributor to physical function, consistent with findings in the general population that HAQ-DI scores increase exponentially with increasing age.^20^ Higher MRSS is also a well-described contributor to poorer physical function in SSc,^11^ and was a determinant of worse disability in our cohort overall as well as those with longer standing disease and lcSSc. Interestingly, higher MRSS scores were not associated with poorer physical function in those with dcSSc in this study, perhaps because these patients had already reached a threshold to confer poorer physical function. The HAQ-DI is very sensitive to hand function,^6^ supported by digital ulcers being significantly associated with poorer physical function. Interestingly, synovitis was not a significant determinant of change in HAQ-DI scores; because arthralgia without synovitis is not collected in the ASCS, the effect of joint pain may have been underestimated. Importantly, patient-reported worsening of both breathlessness and Raynaud’s Phenomenon was associated with increased HAQ-DI scores across all subgroups. This highlights the important role of patient-reported symptoms in identifying important changes in disease activity and severity in SSc.^21^ Raised inflammatory markers are associated with adverse outcomes in SSc and are considered a marker of early, aggressive disease,^22,23^ which fits with our observation that raised CRP was associated with increased HAQ-DI scores in incident disease and dcSSc. Proximal weakness is associated with organ involvement and poorer survival in SSc,^24^ supporting our observed association with impaired physical function. Interestingly, while PAH was associated with poorer physical function in the cohort overall and in those with incident SSc, it was not associated with physical function in prevalent disease, perhaps because patients have gone on to commence treatment with improvement in physical function.^25^ Other cardiopulmonary diseases including ILD and IHD were not associated with impaired physical function in multivariable modelling. This is in keeping with findings from other cohorts, raising questions about the sensitivity of the HAQ-DI to other cardiopulmonary diseases in SSc.^19^ Overall, these data highlight significant and potentially treatable contributors to worse physical function as measured by HAQ-DI scores in SSc across SSc subgroups. In particular, interventions to target skin disease, digital ulcers, Raynaud’s phenomenon, skeletal muscle involvement and dyspnoea may help to prevent worsening of physical function.

The correlates of improvement in HAQ-DI score were less clear. While 71% of participants with a baseline HAQ-DI ⩾ 0.125 units had recorded an improvement in HAQ-DI score, only higher baseline HAQ-DI score was positively associated with this improvement. This finding likely reflects a regression to the mean or floor effect of the HAQ-DI rather than a true clinically meaningful finding. The significant floor effect of the HAQ-DI score has been identified in other rheumatic diseases, with up to one-quarter of patients recording a score of 0.^26^ This may mask the correlates of improvement in HAQ-DI score. In fact, older age and patient-reported dyspnoea were inversely associated with better HAQ-DI scores, indicating that younger patients without breathlessness were more likely to report an improved functional status. Limitations of the content validity of the HAQ-DI in SSc are recognised. The totality of the effects of SSc on daily function may not be adequately captured by the HAQ-DI owing to its emphasis on hand and upper limb function. Despite these limitations, the HAQ-DI has been shown to correlate with clinically important outcomes in SSc.^6,10,11^ Acknowledgement of the limitations of the HAQ-DI has led to the development of SSc-specific instruments of disease impact such as the EULAR Systemic Sclerosis Impact of Disease (ScleroID) questionnaire.^27^ The ScleroID assesses the overall impact of SSc across 10 domains. The ScleroID questionnaire has not been shown to have a significant floor or ceiling effect and may offer better sensitivity to general disease-specific improvement than the HAQ-DI.^27^ However, while the ScleroID is a SSc specific measure of health-related quality of life which correlates with important markers of physical function including 6-min walk testing and hand function performance testing as well HAQ-DI scores, it is not a dedicated measure of daily function. Other measures of physical function including the Patient-Reported Outcomes Measurement System (PROMIS) Physical Function display less of a ceiling effect than the HAQ-DI in individuals with inflammatory myopathy,^28^ although are not SSc specific and lack SSc-specific data about potential floor or ceiling effects and sensitivity to change. Future work is required to develop a SSc-specific measure of daily function, with further testing of newer measures including the PROMIS Physical Function score of benefit in the meantime.

One major limitation of this observational data set is that we could not assess the impact of treatment on outcomes, because there is inherent bias by indication in those who receive treatment. For example, those who receive immunosuppressants to treat severe disease manifestations may be more likely to accrue physical disability. In observational data, there are also likely to be systematic differences between those who do and do not receive treatment with specific manifestations, for example, dcSSc where 81% of individuals received immunosuppressants compared with 19% who did not. Further data are required to better understand what factors are associated with an improvement in physical function in SSc over time, particularly whether specific treatments could improve physical function in prospective, randomised studies. Other limitations of this study include smaller numbers of observations in some subgroups (e.g. dcSSc), which limit the statistical power of some analyses. Furthermore, in the ASCS patient-reported outcome measures including the HAQ-DI are collected during routine outpatient visits. As this study was restricted to participants who had recorded ⩾2 HAQ-DI scores, this cohort may have been biased towards those with milder disease as more unwell participants would be less likely to return serial patient-reported outcome measure surveys. The ASCS is also characterised by a degree of ‘survivor bias’, where sicker individuals are less likely to survive to recruitment. However, despite these limitations, interesting and novel correlates of worsening physical function have been identified between groups.

## Conclusion

Ninety percent of a large SSc cohort recorded clinically meaningful change in physical function over time as measured by HAQ-DI scores. While there were no clinically useful associations of improvement in HAQ-DI score, important correlates of worsening were identified including worsening symptom burden, older age, higher MRSS, raised inflammatory markers and digital ulcers. PAH appears to have a more dominant impact in early disease, while muscle weakness was more prominent in long-standing lcSSc. Further data are required to understand whether effective management of these disease features leads to clinically meaningful improvements in HAQ-DI scores in SSc.

## Supplemental Material

sj-pdf-1-jso-10.1177_23971983251360883 – Supplemental material for Clinical associations of worsening physical function as measured by HAQ-DI scores in systemic sclerosis Supplemental material, sj-pdf-1-jso-10.1177_23971983251360883 for Clinical associations of worsening physical function as measured by HAQ-DI scores in systemic sclerosis by Jessica L Fairley, Dylan Hansen, Susanna Proudman, Joanne Sahhar, Gene-Siew Ngian, Jennifer Walker, Diane Apostolopoulos, Lauren V Host, Wendy Stevens, Mandana Nikpour and Laura Ross in Journal of Scleroderma and Related Disorders
