# Supplementary material for: Clinical associations of worsening physical function as measured by HAQ-DI scores in systemic sclerosis
Source: J Scleroderma Relat Disord. 2025 Aug 19:23971983251360883. Online ahead of print. doi: 10.1177/23971983251360883 (PMC12367724; doi:10.1177/23971983251360883)
Supplement: sj-pdf-1-jso-10.1177_23971983251360883 – Supplemental material for Clinical associations of worsening physical function as measured by HAQ-DI scores in systemic sclerosis [file sj-pdf-1-jso-10.1177_23971983251360883.pdf]

Supplementary Figure S1: Individual plots of HAQ-DI Scores in the cohort overall.

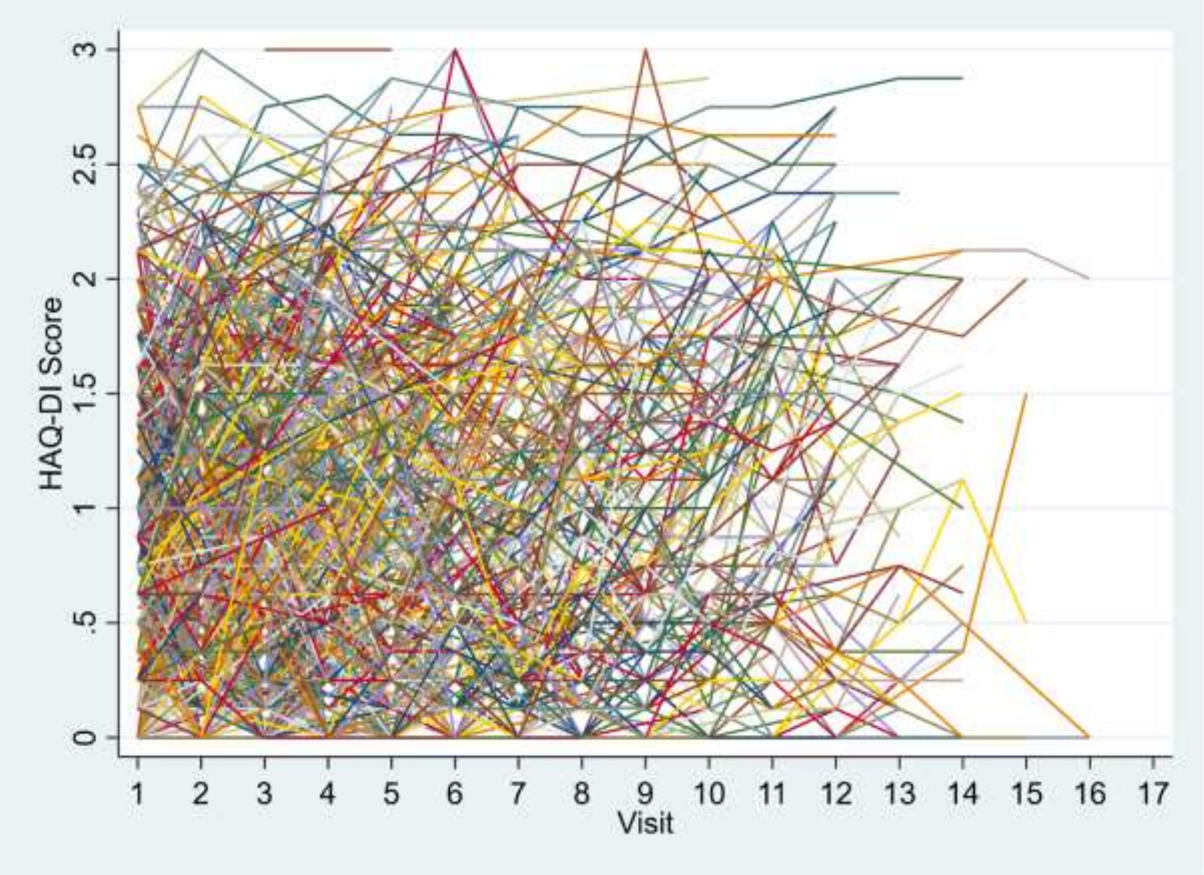

Abbreviations: HAQ-DI (health assessment questionnaire disability index)

Supplementary Table S1: univariable associations of MCID change in HAQ-DI scores at each visit using GEE Regression Modelling

	Worsening				Improvement[9]			
**Variable[1]**	**N^**	**OR**	**95% CI**	**p-value**	**N^**	**OR**	**95% CI**	**p-value**
Baseline HAQ-DI Score[2]	4146	1.3	1.2-1.5	<0.01	2437	1.1	1.0-1.2	0.09
Age at each review 5-year increments	4148	1.1	1.1-1.1	<0.01	2437	1.1	1.0-1.1	<0.01
Female Sex	4148	1.2	1.0-1.5	0.09	2437	1.0	0.9-1.3	0.65
MRSS 5-point increments	3748	1.1	1.1-1.2	<0.01	2283	1.0	1.0-1.1	0.96
Digital Ulcers	4112	1.4	1.2-1.7	<0.01	2427	1.1	1.0-1.3	0.16
Worsening Raynaud's Phenomenon in the last month	4002	1.4	1.2-1.6	<0.01	2373	1.1	1.0-1.3	0.15
Synovitis	4118	1.1	1.0-1.3	0.18	2423	0.9	0.7-1.0	0.03
Proximal muscle weakness[3]	3806	1.7	1.3-2.1	<0.01	2349	1.0	0.9-1.2	0.91
PAH	4148	1.8	1.4-2.2	<0.01	2437	1.5	1.2-1.9	<0.01
ILD[4]	4148	1.2	1.0-1.6	0.09	2437	1.3	0.9-1.7	0.11
ILD Severity[5]								
No ILD		.	.	.		.	.	.
Limited Stage	4123	1.2	0.9-1.7	0.17	2422	1.0	0.7-1.5	0.80
Extensive Stage		1.2	0.8-1.9	0.46		1.5	0.9-2.5	0.14
IHD[6]	4065	1.7	1.3-2.3	<0.01	2408	1.0	0.7-1.4	0.83
Worsening dyspnoea in the last month[7]	3990	1.6	1.4-1.9	<0.01	2359	1.1	0.9-1.3	0.57
CRP>5mg/L	3754	1.6	1.4-1.9	<0.01	2233	1.2	1.0-1.5	0.03
GI symptoms[8]	4084	1.3	1.0-1.6	0.03	2417	1.2	1.0-1.6	0.09

[1]Denotes that item recorded as present at each visit for longitudinal analysis. [2]Baseline HAQ-DI Score defined as first-recorded score. [3]Proximal weakness defined as proximal muscle power of <5/5 on manual muscle testing at each study visit. [4]ILD defined on high-resolution computed tomography of the chest. [5]Extensive ILD defined as >30% HRCT involvement, or 20-30% with percent-predicted FVC<70%. Limited ILD defined as <20% HRCT involvement or 20-30% involvement with FVC≥70%. [6]IHD defined as patient-reported angina, myocardial infarction or abnormal coronary angiogram. [7]Worsening dyspnoea at each study visit defined as patient-reported worsening of breathlessness (yes/no) in month prior to study visit. [8]Gastrointestinal symptoms include dysphagia, reflux, vomiting, gastric antral vascular ectasia, bowel dysmotility, pseudo-obstruction, constipation, faecal incontinence, diarrhoea or bloating. [9]To examine improvement in HAQ-DI scores, of 1117 participants, 290 participants (26%) excluded due to baseline HAQ-DI scores less than 0.125.

Abbreviations: CI (confidence interval), CRP (C-reactive protein), dcSSc (diffuse cutaneous systemic sclerosis), FACIT (functional assessment of chronic illness therapy survey), FVC (forced vital capacity), GEE (generalised estimating equations), GI (gastrointestinal), HAQ-DI (health assessment questionnaire disability index), IHD (ischaemic heart disease), HRCT (high resolution computed tomography), ILD (interstitial lung disease), mg/L (milligrams per litre), MRSS (modified Rodnan Skin Score), N (number), OR (odds ratio), PAH (pulmonary arterial hypertension), SSc (systemic sclerosis).
